# Food insecurity as a barrier to care for people with diabetes in Brazil: gaps and challenges for public health, food, and social protection policies

**DOI:** 10.1590/0102-311XEN054726

**Published:** 2026-10-05

**Authors:** Daniel Ferreira da Silva, Dirce Maria Lobo Marchioni

**Affiliations:** 1 Faculdade de Saúde Pública, Universidade de São Paulo, São Paulo, Brasil.

According to the most recent edition of the *IDF Diabetes Atlas* ^1^, approximately 16.6 million Brazilians had diabetes in 2024, with a projected 24 million cases by 2050, placing the country among those with the highest prevalence in the Americas and ranking sixth worldwide by absolute number of cases. The microvascular complications of diabetes, such as retinopathy, diabetic kidney disease, and neuropathy, and macrovascular complications, such as cardiovascular, cerebrovascular, and peripheral arterial diseases, are major causes of disability, loss of quality of life, and premature mortality in these individuals ^2^. In this context, adequate and healthy eating is recognized as one of the fundamental pillars of glycemic management and the prevention of associated adverse outcomes ^3^.

In Latin American and Caribbean countries, such as Brazil, approximately 80% of the burden of type 2 diabetes mellitus, the most prevalent form of the disease, has been attributed to inadequate diets, characterized mainly by low consumption of whole grains, legumes, fruits, and vegetables ^3^. This scenario reinforces the central role of diet as a modifiable determinant of diabetes risk and management, as well as a strategic target for the development of preventive and care interventions ^4^. However, a substantial portion of the Brazilian population experiences some level of food insecurity ^5^, a condition traditionally measured by the *Brazilian Food Insecurity Scale* (EBIA, acronym in Portuguese), which assesses household perceptions and experiences of worry, uncertainty, or restricted access to food in adequate quantities and of adequate quality, including situations of hunger in the most severe cases ^6^. Food insecurity, therefore, represents a violation of the human right to adequate food ^7^.

In 2022, nearly 59% of Brazilian families experienced some degree of food insecurity ^8^, as a result of the weakening of public food and nutrition policies ^9^ and the worsening of socioeconomic inequalities during the COVID-19 pandemic ^10^. Although this figure has decreased as a result of the resumption and strengthening of public policies on social protection, food, and nutrition, approximately 24.2% of households still experience some level of food insecurity, according to the 2024 *Continuous Brazilian National Household Sample Survey* (Continuous PNAD, acronym in Portuguese) ^5^. Among the groups most affected by this scenario are women, Black people, and individuals with low levels of education and income, who are also among those most affected by the increase in the prevalence of diabetes in the country ^11^ ^,^ ^12^. This overlap of social and economic vulnerabilities increases the challenges of adequately managing the disease, highlighting the need to develop public policies that integrate health interventions, food and nutrition surveillance, and social protection ^13^.

National evidence indicates that food insecurity is associated with reduced dietary diversity and lower consumption of protective foods such as fruits and vegetables ^14^. In addition to providing vitamins, minerals, and fiber - essential components for glycemic control and metabolic health - these foods are important sources of bioactive compounds with recognized antioxidant, anti-inflammatory, and cardioprotective activities that play a fundamental role in preventing microvascular and macrovascular complications associated with diabetes ^15^.

Moreover, in a context of economic constraint, families tend to prioritize low-cost, energy-dense basic and traditional foods over purchasing fruits and vegetables. Data from the latest *Brazilian Household Budget Survey* (POF, acronym in Portuguese) indicated that, among low-income families, cereals, roots, and tubers accounted for approximately 41% of the calories purchased for household consumption, whereas legumes accounted for only 3.6% ^16^. Although these foods are mostly unprocessed or minimally processed, the predominance of carbohydrate-rich food sources, combined with the lower consumption of fiber- and protein-rich foods, may favor dietary patterns with a greater glycemic impact ^17^. In this regard, Brazilian food guidance protocols for people with diabetes have emphasized the importance of meal composition ^18^, including the traditional combination of rice and beans, which may contribute to better glycemic management ^19^.

Although there is no specific diet for people with diabetes, the main guidelines for nutritional management recommend adopting dietary patterns based primarily on whole grains, legumes, fruits, vegetables, and low-fat animal-source foods, such as lean meats and nonfat dairy products ^20^. However, the high cost of these foods in Brazil may represent an additional barrier to diabetes treatment, particularly among socioeconomically vulnerable populations. In this context, national studies have shown that the cost of a healthy diet has become significantly higher than that of dietary patterns based on ultra-processed foods with lower nutritional quality ^21^, especially during extreme climate events and health emergencies that may affect food production, distribution, and prices ^22^ ^,^ ^23^.

Evidence also shows that individuals experiencing food insecurity have lower adherence to recommendations for regular physical exercise and a higher risk of sedentary behaviors ^24^, a relationship that may be explained, in part, by factors such as stress and fatigue, as well as energy-conservation strategies in contexts of food restriction ^25^. Thus, food insecurity represents an obstacle not only to adherence to healthy dietary patterns but also to regular physical exercise, two fundamental pillars of self-care for people with diabetes ^13^.

Moreover, the socioeconomic conditions that often determine food insecurity may compromise adherence to pharmacological treatment for diabetes. Families facing budget constraints frequently need to make financial trade-offs, such as choosing between purchasing food and paying for other basic needs, including medications, transportation, and and housing-related expenses. This instability may reduce the regularity of medication use, increasing the risk of glycemic decompensation and, consequently, chronic complications related to the disease ^13^. This phenomenon may be particularly important in Brazil, as some individuals with diabetes still incur direct out-of-pocket expenses for medications, despite the existence of public pharmaceutical assistance policies ^26^. Data from the latest *Brazilian National Health Survey* (PNS, acronym in Portuguese), for example, indicated that only 51.5% of individuals with diabetes reported obtaining medications through the Brazilianj Popular Pharmacy Program ^27^.

Despite the relevance of the topic, public policies aimed at caring for people with diabetes in Brazil still insufficiently address the role of food security in successful treatment ^28^ ^,^ ^29^ ^,^ ^30^. Only recently was the Food Insecurity Risk Screening (TRIA, acronym in Portuguese) incorporated into primary health care (PHC), the main entry point to the Brazilian Unified National Health System (SUS, acronym in Portuguese) for the follow-up of people with chronic diseases ^31^. In addition, the role of food insecurity in diabetes-related health outcomes remains absent from the main Brazilian clinical guidelines and normative instruments, such as clinical protocols and therapeutic guidelines ^28^ ^,^ ^29^. This gap reinforces the need to incorporate guidance that considers the social determinants of health and the reality of people in situations of socioeconomic vulnerability into these documents ^32^.

While food insecurity is insufficiently addressed in the main Brazilian clinical guidelines for diabetes management, international documents already recognize this problem as an important barrier to achieving therapeutic goals. The American Diabetes Association (ADA), for example, highlights food insecurity as a factor that may hinder treatment adherence, self-care, and glycemic control, recommending that health professionals assess this condition among their patients ^33^. Similarly, the International Society for Pediatric and Adolescent Diabetes (ISPAD) recognizes food insecurity as a social determinant that may compromise diabetes management in children and adolescents, especially in low- and middle-income countries characterized by pronounced social inequalities ^34^.

Given the limitations imposed by food insecurity, it is essential that nutritional recommendations for people with diabetes be adapted to the socioeconomic and cultural realities of the populations served ^35^. Strategies such as prioritizing regional foods and lower-cost seasonal fruits and vegetables ^36^, as well as promoting traditional dishes with more favorable nutritional profiles and providing guidance on food combinations that reduce glycemic impact, may be viable alternatives for improving glycemic management in these individuals ^35^ ^,^ ^37^.

Addressing food insecurity in the care of people with diabetes also requires integrating therapeutic care with food security policies and social protection programs ^33^ ^,^ ^34^. Among the immediate response measures, the expansion of direct cash transfer programs - preferably with benefit amounts adjusted to the cost of a healthy diet - stands out, alongside the strengthening of public food and nutrition security facilities, such as community and solidarity kitchens and popular restaurants, so that they prioritize menus appropriate for people with chronic conditions ^38^. It is also essential to expand the supply and availability of fresh foods through family farming, community gardens, and local farmers’ markets, especially in areas with limited availability of these foods ^36^ ^,^ ^39^.

In this context, the Brazilian National Strategy for Food and Nutritional Security in Cities, established by *Decree n. 11,822* of December 12, 2023 ^40^, represents an important government initiative aimed at expanding the availability of and access to healthy foods in urban centers, especially among socially vulnerable populations. In addition, the establishment of the New National Basic Food Basket represents a relevant advance by recognizing unprocessed or minimally processed foods as central elements in promoting the human right to adequate food and prohibiting the inclusion of ultra-processed foods in the basic food basket ^41^. More than a reference for food procurement, the new basic food basket serves as a policy instrument to promote public policies aimed at reducing the price of healthy foods through tax incentives, public procurement, and other regulatory strategies ^42^.

PHC, which is fundamental to care coordination and continuity of care within the SUS ^43^, should be integrated with social assistance services so that the identification of people and families in vulnerable situations through the TRIA results in timely access to social protection measures ^31^. The effectiveness of this strategy depends not only on identifying food insecurity but also on the existence of integrated care pathways capable of ensuring continuous follow-up and intersectoral care. In this context, *Interministerial Ordinance n. 25/2023* ^44^ of the Brazilian Ministry of Development and Social Assistance, Family, and Fight Against Hunger/Brazilian Ministry of Health represents an important advance by establishing guidelines for integrating social assistance, health, and food security actions, strengthening the identification, follow-up, and care of populations in situations of socioeconomic vulnerability.

Therefore, advancing the integration of the food security and diabetes management agendas is essential to improve the quality of care, reduce inequalities, and ensure better living conditions for the most vulnerable populations ^33^. Incorporating routine assessment of food insecurity into health services, strengthening intersectoral actions, and ensuring access to adequate and healthy foods should be viewed as strategic components of the response to the increasing prevalence of diabetes in Brazil.

## Data Availability

Not applicable.
