# Supplementary material for: Food insecurity as a barrier to care for people with diabetes in Brazil: gaps and challenges for public health, food, and social protection policies
Source: Cad Saude Publica. 2026 Oct 5;42:e00054726. doi: 10.1590/0102-311XEN054726 (PMC13640033; doi:10.1590/0102-311XEN054726)
Supplement: Translation

# Translation: Insegurança alimentar como barreira ao cuidado de pessoas com diabetes no Brasil: lacunas e desafios para as políticas públicas de saúde, alimentação e proteção social

**DOI:** 10.1590/0102-311XPT054726

**Authors:** Daniel Ferreira da Silva, Dirce Maria Lobo Marchioni

**Affiliations:** 1 Faculdade de Saúde Pública, Universidade de São Paulo, São Paulo, Brasil.

De acordo com a edição mais recente do *IDF Diabetes Atlas* ^1^, cerca de 16,6 milhões de brasileiros possuíam diabetes em 2024, com projeção para 2050 de 24 milhões de casos, o que posiciona o país entre os de maior prevalência nas Américas e em sexto lugar no ranking mundial em números absolutos. As complicações microvasculares decorrentes do diabetes, como a retinopatia, a doença renal do diabetes e a neuropatia, e macrovasculares, como as doenças cardiovasculares, cerebrovasculares e arteriais periféricas, representam importantes causas de incapacidade, perda de qualidade de vida e mortalidade precoce nesses indivíduos ^2^. Nesse contexto, a alimentação adequada e saudável é reconhecida como um dos pilares fundamentais para o manejo glicêmico e a prevenção de desfechos adversos associados ^3^.

Em países da América Latina e do Caribe, como o Brasil, cerca de 80% da carga de diabetes mellitus tipo 2, a forma mais prevalente da doença, tem sido atribuída a dietas inadequadas, caracterizadas principalmente pelo baixo consumo de cereais integrais, leguminosas, frutas, legumes e verduras ^3^. Esse cenário reforça o papel central da alimentação como determinante modificável do risco e do manejo do diabetes, bem como alvo estratégico para o desenvolvimento de ações preventivas e de cuidado ^4^. No entanto, uma parcela importante da população brasileira vivencia algum nível de insegurança alimentar ^5^, condição tradicionalmente aferida pela *Escala Brasileira de Insegurança Alimentar* (EBIA), que avalia a percepção e a experiência domiciliar de preocupação, incerteza ou restrição no acesso a alimentos em quantidade e qualidade adequadas, incluindo situações de fome nos casos mais graves ^6^. A insegurança alimentar, portanto, representa uma violação do direito humano à alimentação adequada (DHAA) ^7^.

Em 2022, quase 59% das famílias brasileiras vivenciavam algum grau de insegurança alimentar ^8^, como resultado do enfraquecimento de políticas públicas de alimentação e nutrição ^9^ e do agravamento das desigualdades socioeconômicas durante a pandemia de COVID-19 ^10^. Embora esse número tenha reduzido, como resultado da retomada e do fortalecimento de políticas públicas de proteção social, alimentação e nutrição, cerca de 24,2% dos domicílios ainda permanecem em algum nível de insegurança alimentar de acordo com a *Pesquisa Nacional por Amostra de Domicílios Contínua* (PNAD-Contínua) de 2024 ^5^. Entre os grupos mais afetados por esse cenário estão as mulheres, pessoas negras e indivíduos com baixa escolaridade e renda, que também figuram entre os mais impactados pelo aumento da prevalência de diabetes no país ^11^ ^,^ ^12^. Essa sobreposição de vulnerabilidades sociais e econômicas amplia os desafios para o manejo adequado da doença, evidenciando a necessidade de desenvolvimento de políticas públicas que integrem ações de saúde, vigilância alimentar e nutricional, e proteção social ^13^.

Evidências nacionais indicam que a insegurança alimentar está associada à redução da diversidade da dieta, com menor consumo de alimentos protetores como frutas, legumes e verduras ^14^. Esses alimentos, além de fornecerem vitaminas, minerais e fibras, componentes essenciais para o controle glicêmico e a saúde metabólica, são importantes fontes de compostos bioativos com reconhecida ação antioxidante, anti-inflamatória e cardioprotetora que desempenham papel fundamental na prevenção de complicações microvasculares e macrovasculares associadas ao diabetes ^15^.

Além disso, em um contexto de restrição econômica, as famílias tendem a priorizar alimentos básicos e tradicionais de baixo custo e elevada densidade energética em detrimento da aquisição de frutas, legumes e verduras. Dados da última *Pesquisa de Orçamentos Familiares* (POF) indicaram que, entre as famílias de baixa renda, cereais, raízes e tubérculos responderam por cerca de 41% das calorias adquiridas para o consumo domiciliar, e as leguminosas corresponderam a apenas 3,6% ^16^. Embora esses alimentos sejam, em sua maioria, *in natura* ou minimamente processados, a predominância de fontes alimentares ricas em carboidratos, associada à menor participação de alimentos fontes de fibras e proteínas, pode favorecer padrões alimentares de maior impacto glicêmico ^17^. Nesse sentido, protocolos brasileiros de orientação alimentar para pessoas com diabetes têm enfatizado a importância da composição das refeições ^18^, a exemplo da combinação tradicional de arroz e feijão que pode contribuir para um melhor manejo glicêmico ^19^.

Embora não exista uma dieta específica para pessoas com diabetes, as principais diretrizes de manejo nutricional recomendam a adoção de padrões alimentares baseados, principalmente, no consumo de cereais integrais, leguminosas, frutas, legumes, verduras e produtos de origem animal com baixo teor de gordura, como carnes magras e laticínios desnatados ^20^. No entanto, o custo elevado desses alimentos no Brasil pode representar uma barreira adicional ao tratamento do diabetes, especialmente se tratando de populações em situação de vulnerabilidade socioeconômica. Nesse aspecto, estudos nacionais têm demonstrado que o custo de uma alimentação saudável tem se tornado significativamente mais alto quando comparado a padrões alimentares baseados em alimentos ultraprocessados e de menor qualidade nutricional ^21^, sobretudo durante eventos climáticos extremos e emergências sanitárias que podem afetar a produção, a distribuição e os preços dos alimentos ^22^ ^,^ ^23^.

Evidências também demonstram que indivíduos em situação de insegurança alimentar apresentam menor adesão às recomendações de prática regular de exercício físico e maior risco de comportamentos sedentários ^24^, relação que pode ser explicada, em parte, por fatores como estresse e fadiga, além de estratégias de preservação de energia em contextos de restrição alimentar ^25^. Dessa forma, a insegurança alimentar representa um obstáculo não apenas à adesão a padrões alimentares saudáveis, mas também à prática regular de exercício físico, dois pilares fundamentais do autocuidado para pessoas com diabetes ^13^.

Além do mais, as condições socioeconômicas que frequentemente determinam a insegurança alimentar podem comprometer a adesão ao tratamento farmacológico do diabetes. Famílias em situação de restrição orçamentária frequentemente precisam realizar compensações financeiras, como escolher entre a compra de alimentos e o pagamento de outras necessidades básicas, incluindo medicamentos, transporte e despesas relacionadas à moradia. Essa instabilidade pode reduzir a regularidade do uso de medicamentos, favorecendo a ocorrência de descompensações glicêmicas e, consequentemente, de complicações crônicas relacionadas à doença ^13^. Esse fenômeno pode ser particularmente importante no Brasil, uma vez que parte dos indivíduos com diabetes ainda incorre em gastos diretos com medicamentos, apesar da existência de políticas públicas de assistência farmacêutica ^26^. Dados da última *Pesquisa Nacional de Saúde* (PNS), por exemplo, indicaram que apenas 51,5% dos indivíduos com diabetes relataram obter medicamentos por meio do Programa Farmácia Popular ^27^.

Apesar da relevância do tema, as políticas públicas voltadas ao cuidado da pessoa com diabetes no Brasil ainda abordam de forma insuficiente a dimensão da segurança alimentar para o sucesso do tratamento ^28^ ^,^ ^29^ ^,^ ^30^. Apenas recentemente foi incorporada a Triagem de Risco de Insegurança Alimentar (TRIA) na atenção primária à saúde (APS), principal porta de entrada do Sistema Único de Saúde (SUS) brasileiro para o acompanhamento de pessoas com doenças crônicas ^31^. Além disso, a discussão sobre o papel da insegurança alimentar nos resultados de saúde relacionados ao diabetes permanece ausente das principais diretrizes clínicas e instrumentos normativos brasileiros, como os protocolos clínicos e diretrizes terapêuticas ^28^ ^,^ ^29^. Essa lacuna reforça a necessidade de incorporar orientações que considerem os determinantes sociais de saúde e a realidade das pessoas em situação de vulnerabilidade socioeconômica nesses documentos ^32^.

Enquanto no Brasil a insegurança alimentar é abordada de forma insuficiente nas principais diretrizes clínicas para o manejo do diabetes, documentos internacionais já reconhecem esse problema como uma barreira importante para o alcance das metas terapêuticas. A Associação Americana de Diabetes (ADA, acrônimo em inglês), por exemplo, destaca a insegurança alimentar como um fator que pode dificultar a adesão ao tratamento, o autocuidado e o controle glicêmico, recomendando que profissionais de saúde avaliem essa condição entre os seus pacientes ^33^. Da mesma forma, a Sociedade Internacional de Diabetes Pediátrico e do Adolescente (ISPAD, acrônimo em inglês) reconhece a insegurança alimentar como um determinante social que pode comprometer o manejo do diabetes em crianças e adolescentes, especialmente em países de baixa e média renda, profundamente marcados por desigualdades sociais ^34^.

Diante das limitações impostas pela insegurança alimentar, é fundamental que as recomendações nutricionais para pessoas com diabetes, portanto, sejam adaptadas à realidade socioeconômica e cultural das populações atendidas ^35^. Estratégias como a priorização de alimentos regionais e de frutas, legumes e verduras sazonais, de menor custo ^36^, bem como a valorização de preparações tradicionais com melhor perfil nutricional e orientações sobre combinações alimentares que reduzam o impacto glicêmico, podem ser alternativas viáveis para a melhora do manejo glicêmico nesses indivíduos ^35^ ^,^ ^37^.

O enfrentamento da insegurança alimentar no cuidado de pessoas com diabetes também requer a articulação da assistência terapêutica com políticas de segurança alimentar e programas de proteção social ^33^ ^,^ ^34^. Entre as medidas de resposta imediata, destacam-se a ampliação dos programas de transferência direta de renda, de preferência, com valores ajustados ao custo de uma dieta saudável; e a qualificação de equipamentos públicos de segurança alimentar e nutricional, como cozinhas comunitárias e solidárias e restaurantes populares, para que priorizem cardápios adequados a pessoas com condições crônicas ^38^. Também é fundamental que sejam ampliado o abastecimento e a oferta de alimentos frescos por meio da agricultura familiar, hortas comunitárias e feiras de produtores locais, especialmente em áreas de baixa disponibilidade desses alimentos ^36^ ^,^ ^39^.

Nesse contexto, a Estratégia Alimenta Cidades, instituída pelo *Decreto nº 11.822*, de 12 de dezembro de 2023 ^40^, se caracteriza como uma importante iniciativa governamental voltada à ampliação da disponibilidade e do acesso a alimentos saudáveis nos centros urbanos, especialmente entre as populações em situação de vulnerabilidade social. Além do mais, a instituição da Nova Cesta Básica Nacional de Alimentos representa um avanço relevante, ao reconhecer os alimentos in natura ou minimamente processados como elementos centrais para a promoção do DHAA e vedar a presença dos ultraprocessados entre os alimentos que compõem a cesta básica ^41^. Mais do que uma referência para a aquisição de alimentos, a nova cesta básica atua como um instrumento indutor de políticas públicas voltadas à redução do preço de alimentos saudáveis, por meio de incentivos fiscais, compras públicas e outras estratégias regulatórias ^42^.

A APS, fundamental para a coordenação do cuidado e continuidade da assistência no SUS ^43^, deve ser articulada aos serviços de assistência social, de modo que a identificação de pessoas e famílias em situação de vulnerabilidade por meio da TRIA resulte em acesso oportuno a ações de proteção social ^31^. A efetividade dessa estratégia depende não apenas da identificação da insegurança alimentar, mas da existência de fluxos assistenciais integrados capazes de garantir acompanhamento contínuo e atenção intersetorial. Nesse contexto, a *Portaria Interministerial nº 25/2023* ^44^, do Ministério do Desenvolvimento e Assistência Social, Família e Combate à Fome/Ministério da Saúde, representa um importante avanço ao estabelecer orientações para a integração das ações de assistência social, saúde e segurança alimentar, fortalecendo a identificação, o acompanhamento e o cuidado de populações em situação de vulnerabilidade.

Avançar, portanto, na integração entre as agendas de segurança alimentar e manejo do diabetes é essencial para qualificar o cuidado, reduzir as desigualdades e garantir melhores condições de vida para as populações mais vulneráveis ^33^. Incorporar a avaliação rotineira da insegurança alimentar nos serviços de saúde, fortalecer as ações intersetoriais e garantir o acesso a alimentos adequados e saudáveis devem ser vistos como componentes estratégicos na resposta ao aumento da prevalência de diabetes no Brasil.

## Data Availability

Não se aplica.
